# Design and development of an open-source framework for citizen-centric environmental monitoring and data analysis

**DOI:** 10.1038/s41598-022-18700-z

**Published:** 2022-08-24

**Authors:** Sachit Mahajan

**Affiliations:** Computational Social Science, ETH Zurich, 8092 Zürich, Switzerland

**Keywords:** Environmental social sciences, Environmental sciences

## Abstract

Cities around the world are struggling with environmental pollution. The conventional monitoring approaches are not effective for undertaking large-scale environmental monitoring due to logistical and cost-related issues. The availability of low-cost and low-power Internet of Things (IoT) devices has proved to be an effective alternative to monitoring the environment. Such systems have opened up environment monitoring opportunities to citizens while simultaneously confronting them with challenges related to sensor accuracy and the accumulation of large data sets. Analyzing and interpreting sensor data itself is a formidable task that requires extensive computational resources and expertise. To address this challenge, a social, open-source, and citizen-centric IoT (Soc-IoT) framework is presented, which combines a real-time environmental sensing device with an intuitive data analysis and visualization application. Soc-IoT has two main components: (1) CoSense Unit—a resource-efficient, portable and modular device designed and evaluated for indoor and outdoor environmental monitoring, and (2) exploreR—an intuitive cross-platform data analysis and visualization application that offers a comprehensive set of tools for systematic analysis of sensor data without the need for coding. Developed as a proof-of-concept framework to monitor the environment at scale, Soc-IoT aims to promote environmental resilience and open innovation by lowering technological barriers.

## Introduction

Over the past years, the world has seen massive growth in urbanization at regional and national levels. Although rapid urbanization has led to economic growth, it has led to environmental degradation as well^1^. Activities like excessive use of fossil fuels for energy production and deforestation to create more urban spaces are already contributing to the degradation of air quality. Traffic related pollution (mostly air and noise pollution) is also contributing to environment and health degradation. The World Health Organization (WHO) has already identified traffic-related noise as a public health risk since it can disrupt the human sleep cycle, increase stress, and lead to psychiatric disorders^2^. Noise and air pollution aren’t the only side effects of urbanization. Excessive use of artificial lighting in cities already contributes to light pollution, which leads to the release of more heat into the atmosphere^3^. The environmental pollution is not only limited to developing or under-developed countries, but even high-income countries are getting adversely affected by it^4^. According to a report by WHO^5^, indoor and outdoor air pollution exposure is strongly linked to heart and cardiovascular diseases. Among different pollutants, particulate matter (PM) is known to be more dangerous for human health as compared to gaseous components^6^. While there have been numerous efforts by governments and environmental protection agencies to combat the threats like air pollution, there has been limited success in a reduction in the levels of pollutants like PM. This has been mainly due to the limited availability of accurate and fine-grained air quality data to create effective policies. The official monitoring networks used in most countries around the world comprise a limited number of fixed monitoring stations. They are accurate but only covered a limited geographical area^7^. Due to the expensive and bulky nature of such stations, it is not logistically possible to do a mass deployment of such stations. Similarly, noise monitoring infrastructures are limited to to the expensive nature of professional sound level meters and poorly calibrated low-cost noise monitoring sensors^8^.

It is now easier than ever to collect large-scale environmental data, thanks to the rise of the smart city concept. Smart cities are cities in which information and communication technology (ICT) is incorporated into the city fabric to collect data for the purpose of upgrading infrastructure and providing better services to people^9^. One of the strategic aims of smart cities has been to improve economic, social and environmental sustainability^10^. To fulfil the sustainability needs, smart cities use technologies that improve the quality of life, improve urban operations and services and promote sustainable development^11^. The Internet of Things (IoT) is already speeding up smart city innovation by allowing complicated systems such as traffic control, environmental monitoring and automatic street lighting to be managed using data from networked sensors. The usage of IoT devices embedded with low-cost sensors for environmental monitoring has increased dramatically in recent years^12^. Because of the low cost of the sensors, citizens have been able to access these technologies and use them for activities like crowd-sensing, in which a group of residents uses low-cost sensing systems to monitor the surroundings and collect actionable data. The open-source nature of environmental monitoring solutions has also contributed to the rise in IoT-based environmental monitoring. Open-source refers to any program or platform whose source code is freely available and can be reused and redistributed^13^. The application of crowd-based methods in research has substantially increased over the past decade^10^. This has resulted in an increase in various forms of crowd involvement, such as crowdsourcing and Citizen Science; the former involves people in gathering ideas and solutions to various problems^10^, while the latter allows people to participate in scientific processes and provide input and valuable contributions^14^. Low-cost IoT systems have enabled large-scale deployments and data collection at finer spatio-temporal resolutions, which were previously impossible with traditional monitoring systems due to logistical and financial constraints^15^. These devices provide real-time air quality data that can be useful for understanding the ambient environment and assisting decision-makers in making better policies for pollution control. There have been several examples of how low-cost environmental monitoring solutions have been implemented around the world to raise air pollution awareness^15–17^, create air pollution data sets^15,18^, promote citizen participation in air quality monitoring^19–21^, and create applications for data-informed decision making^22,23^. The impact is not limited to raising awareness, but also to developing innovative methodologies and technologies to improve citizens’ well-being^24^. The studies by Pigliautile et al.^25,26^ are good examples of how innovative solutions like wearable sensing technology can be used to investigate complex topics like microclimate variations and pedestrian comfort. The valuable data crowdsourced through IoT has a direct impact on the location-based services provided to citizens. The data is instrumental in creating advanced air quality data analysis frameworks^27,28^, PM2.5 forecasting systems^29–31^, ecosystems for smart environment governance^32,33^, and resilient cities^34^.

**Figure 1:**
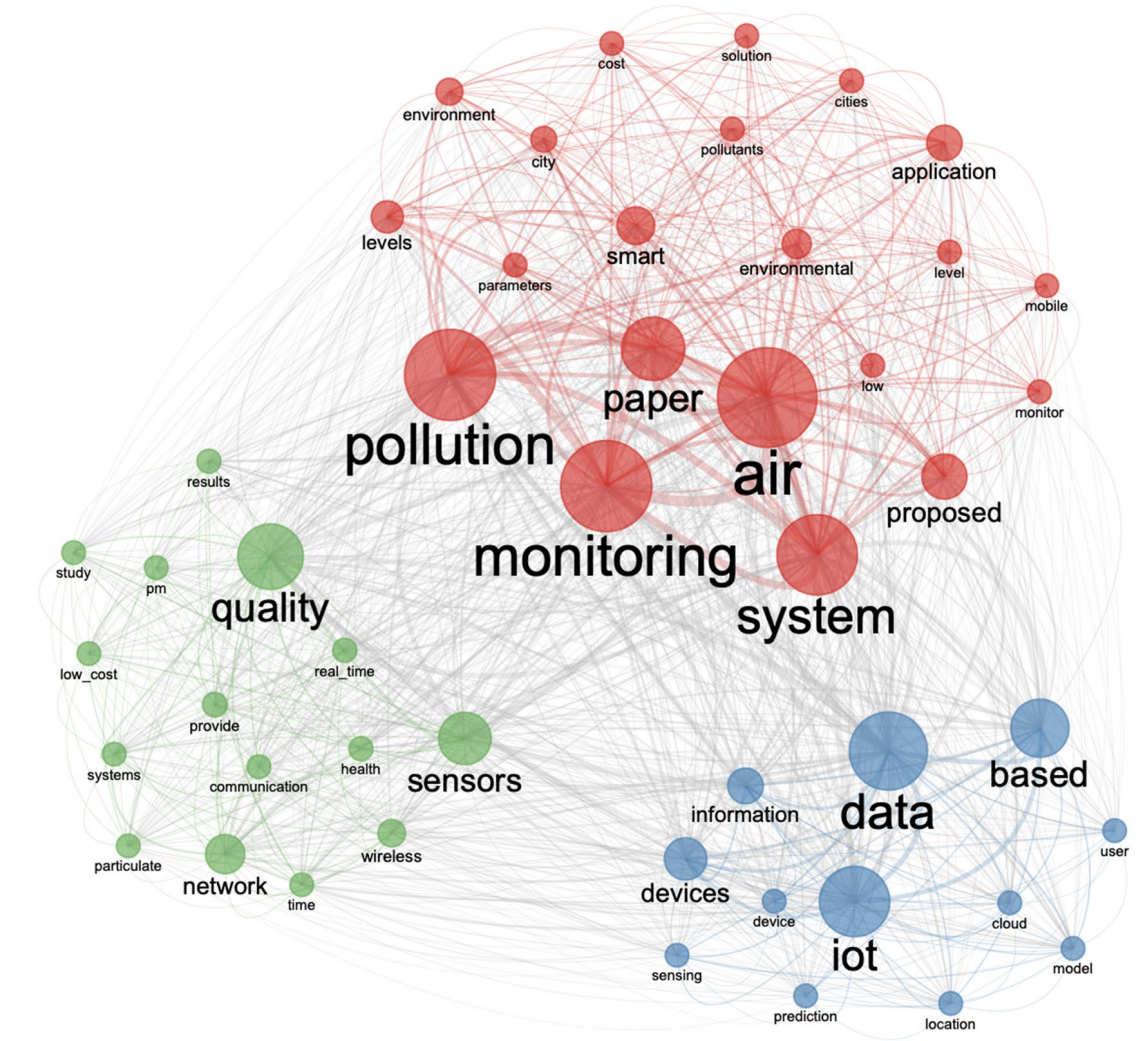
Network visualization of frequently occurring terms within the existing literature related to keywords “Internet of Things” and “Air pollution monitoring”.

*Bibliometric analysis* Bibliometric analysis is an efficient method to understand research trends and scholarly networks in different disciplines^28^. In this work, bibliometric analysis has been carried out to highlight the state of the art as well as research gaps. To understand how the keywords like “Internet of Things” and “Air pollution monitoring”have been used within the existing literature and in what context, quantitative bibliometric analysis and knowledge mapping approaches were used. The keyword co-occurrence method was used to find the keywords that are discussed more frequently together. To perform the analysis, first, a search query was created that searched all the papers indexed in the Web of Science database^35^ since the year 1990 containing the topics “Internet of Things”AND “Air pollution monitoring”. The search included the title of the paper, abstract and author’s keywords. The search query resulted in 65 papers. The data from those 65 papers were used to create the keyword co-occurrence network graph, shown in Fig. 1. bibliometrix package of R was used to perform the network analysis^36^. Keyword co-occurrence analysis was used to create the network graph by looking for very frequent terms in the database created by the initial search query. The nodes were chosen from the top 50 most frequently occuring terms. There were at least two edges on each node. To detect the communities in the network, the Louvain method of community detection was implemented^37^. It is a clustering algorithm that is based on the greedy approach to modularity optimization. In the beginning, every node is assigned to a unique cluster. This is followed by placing each node is into another cluster to make the network more modular. The process is repeated several times until there is no further scope for improving the network modularity. It can be observed in Fig. 1 that there are three key research clusters. The largest cluster is mainly focused on air pollution monitoring systems, the environment, and smart cities. Between the other two clusters, one focuses on the IoT devices, data, and information while the other is more centered around PM, networks, and sensors. Despite a strong focus of existing research on IoT systems, environment, data, and cities, surprisingly there was no mention of keywords like ‘citizens’, ‘community’, ‘open-source’, or ‘sustainability’. There is a clear gap when it comes to bridging the IoT, environmental monitoring, citizen participation, and open-source solutions. This reinforces the relevance of this study which aims at creating a proof-of-concept framework for environmental monitoring that is citizen-centric, open-source, and sustainable.

**Figure 2:**
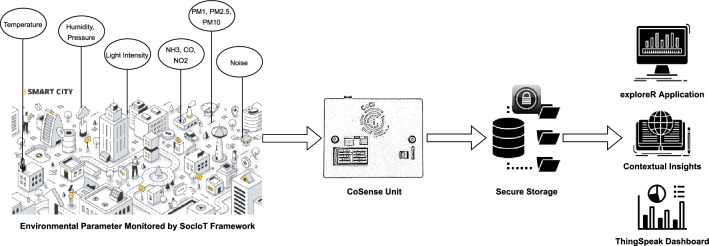
Overview of the proposed framework.

*Motivation* While the use of low-cost sensors has improved the air quality data availability and access, several challenges still need to be addressed. Data quality and accuracy of low-cost sensors remains one of the key challenges^38–40^. It has been widely discussed how an IoT application could be considered useless due to poor sensor data quality^41^. This not only restricts the potential use of IoT data for various applications but also creates an environment where the acceptability of citizen-generated data reduces due to a lack of accuracy. This makes it imperative that the hardware and software components of the IoT framework are extensively validated to successfully handle the sensor data with minimum errors and missing data. It has also been observed that IoT systems are sometimes designed in less human-centric ways^42^. This can be related to highly automated sensors, black-box algorithms, data accessibility, and complex data analysis tools. The lack of value-sensitive design often results in user disempowerment followed by disengagement^43,44^. This is a critical concern as the majority of citizen science air quality monitoring projects depend on volunteers who are investing their time and resources. For example, in many citizen science air quality monitoring projects, the citizens rely on experts to do the data analysis and interpretation. Though scientific expertise is needed to analyze data but creating opportunities for citizens to do data analysis and interpretation allows bridging the gap between experts and non-experts. It also fosters a sense of collaboration and trust that are important for successfully doing citizen science. Another pressing issue is the consideration of sustainability factors for the design, development, and implementation of low-cost sensor systems. Based on a study^45^, it was found that there is limited literature when it comes to understanding the long-term sustainability of low-cost sensor solutions for environmental monitoring. The predominant focus of most of the studies has been on data collection and analysis. This could be partly because most of these sensor studies are conducted in regions that have significant resources and infrastructure^45^.

This paper addresses these challenges by describing the design, implementation, and potential impact of a social, open-source, and citizen-centric IoT (Soc-IoT, pronounced as *‘Society’*) framework Fig. 2. Soc-IoT is proposed as an open-source^46^ environmental monitoring and data analysis framework that encourages collective and participatory action as well as social impact. It comprises of two key components that are specifically designed and developed to address the issues raised previously in the paper. The first component is the CoSense Unit which is a modular and open-source environment sensing device that can provide consistent and reliable air quality data. It has been thoroughly tested and validated in a real-world setting and evaluated by co-locating with a Swiss government environmental monitoring station. The carbon footprint and energy usage of these low-cost gadgets are also examined to determine the CoSense Unit’s environmental sustainability. The framework’s second core component is the exploreR, an open-source RShiny-based data analysis and visualization application. The app is intended to lower technological obstacles, particularly those connected to programming, by allowing citizens and specialists alike to examine and interpret sensor data in a useful way. To address the critical issue of collaborative environmental sensing, the entire framework is designed to establish an innovative ecosystem that encourages cooperation, sustainable practices, and inclusivity.

## Methods

This section describes the methodology behind the design of the proposed Soc-IoT framework. The following paragraphs provide a detailed overview of the system architecture, sensor prototype, and data analysis application.

**Figure 3:**
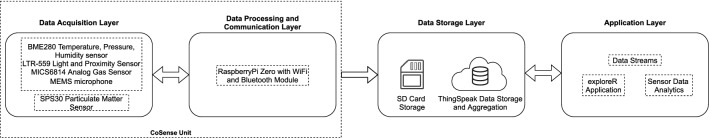
Soc-IoT system infrastructure.

### System architecture

The Soc-IoT framework is based on the principle of open-source hardware and software. Figure 3 shows the system architecture of the proposed framework. It comprises four major components:

- Data Acquisition Layer: This layer consists of the sensors that are responsible for sensing the environmental variables monitored by the CoSense Unit. The current version of the CoSense Unit consists of a Sensirion SPS 30 PM sensor that can sense PM1, PM2.5, and PM10. The Enviro+ board for Raspberry Pi is used to monitor temperature, pressure, humidity, light intensity, noise, and gas concentration (NO2, NH3, and CO). As the codes for these sensors are open-source, the users can easily reprogram the sensors based on their requirements as well as examine and verify the sensors without any complications. More details about the hardware components are available in the next section.
- Data Processing and Communication Layer: This layer is responsible for processing and integrating data from different sensors and communicating it to the data storage layer. A Raspberry Pi Zero handles all the functions related to data processing and communication. The Wi-Fi module of Raspberry Pi Zero is used to create an access point that allows a continuous flow of data from the Raspberry Pi Zero to the data storage layer. Different data transmission protocols were considered for data transmission. The current version of the CoSense Unit uses the Hyper Text Transfer Protocol (HTTP) due to its high transmission reliability and infrastructure^40,47^.
- Data Storage Layer: This layer is responsible for securely storing the data. The current version of the framework allows two storage options. Either the data can be directly transmitted to the ThingSpeak database or the user can save the data locally on the SD card that comes with the Raspberry Pi. This is beneficial in case of unavailability of the internet to send the data to the ThingSpeak cloud. The users can simply upload the data from the SD card to their data stream at a later stage. This also provides more control to the users over their data. If the users prefer not to share their data, they can opt out of making their data stream public and use the data from the stream and the SD card for their information.
- Application Layer: The data from the storage layer is used to create applications that are used to make sense of the raw data. This includes data streams, visualizations, and data analysis applications. The Soc-IoT framework includes two core applications: (1) ThingSpeak dashboard that allows a user to create data streams, visualize data, and use Matlab functions to perform data analysis. (2) An R-based application that allows a user to do data processing, analysis, visualization, and performs Machine Learning (ML) on the data. Section 3 includes more details about the applications.

### Hardware implementation

Despite the fact that the quality of one’s environment has a significant impact on one’s health, most people are unaware of it^48^. The majority of harmful pollutants, for example, are colorless and odorless, making it difficult to determine their actual levels. As a result, having an efficient system that quantifies pollution levels and provides feedback is critical. Objective measurements and easily understood visualizations could assist people in consciously processing - and, if necessary, adjusting - air quality, lighting, and noise levels. In other words, objective measurements are required to induce behavioral change. The CoSense Unit is the hardware component of the Soc-IoT framework that is responsible for indoor and outdoor environmental monitoring. It has been designed using state-of-the-art sensors and a single board computer. The current version of the CoSense Unit measures: (1) PM concentration in the air; (2) temperature, pressure, humidity; (3) gas (NO2, NH3, CO) concentration; (3) light intensity; and (4) noise. The modular nature of the device allows users to easily remove and add more sensors based on their requirements. The CoSense Unit is easy to assemble and can be used for indoor and outdoor environment sensing. For building a participatory sensing unit, it is important to select the most suitable sensors. While there are a lot of low-cost sensors circulating in the market, not all of them are accurate and efficient when it comes to long-term environmental monitoring. For PM monitoring, the CoSense Unit uses a Sensirion SPS30 PM sensor. The sensor was selected because of its high precision, accuracy, and low bias as compared to other available PM sensors like Plantower PMS5003, SM-UART-04L PM sensor^49,50^. The SPS30 is capable of monitoring PM1, PM2.5, PM4, and PM10 using the light-based scattering principle. The current version of the CoSense Unit is programmed to monitor PM1, PM2.5, and PM10. In addition to the SPS30 sensor, a sensor array called Enviro Plus that has sensors like BME280 (temperature, humidity, pressure), MICS6814 analog gas sensor (NO2, NH3, and CO), LTR-559 light and proximity sensor, and a MEMS microphone (noise) is also added to the CoSense Unit. It also includes the ADS1015 analog to digital converter for converting data from the analog gas sensor and a color LCD. The data produced by the analog gas sensor is in kOhms, which is not the standard unit for gas concentration monitoring. The sensor program converts it into parts per million (ppm) to get an indicative value. Due to a lot of conversion processes, it is difficult to precisely validate it with a regulatory or industry-grade monitor. Nevertheless, the values from the gas sensor can be used as indicative values for understanding how the concentration is changing in a given environment, as highlighted by many studies^51,52^.

**Figure 4:**
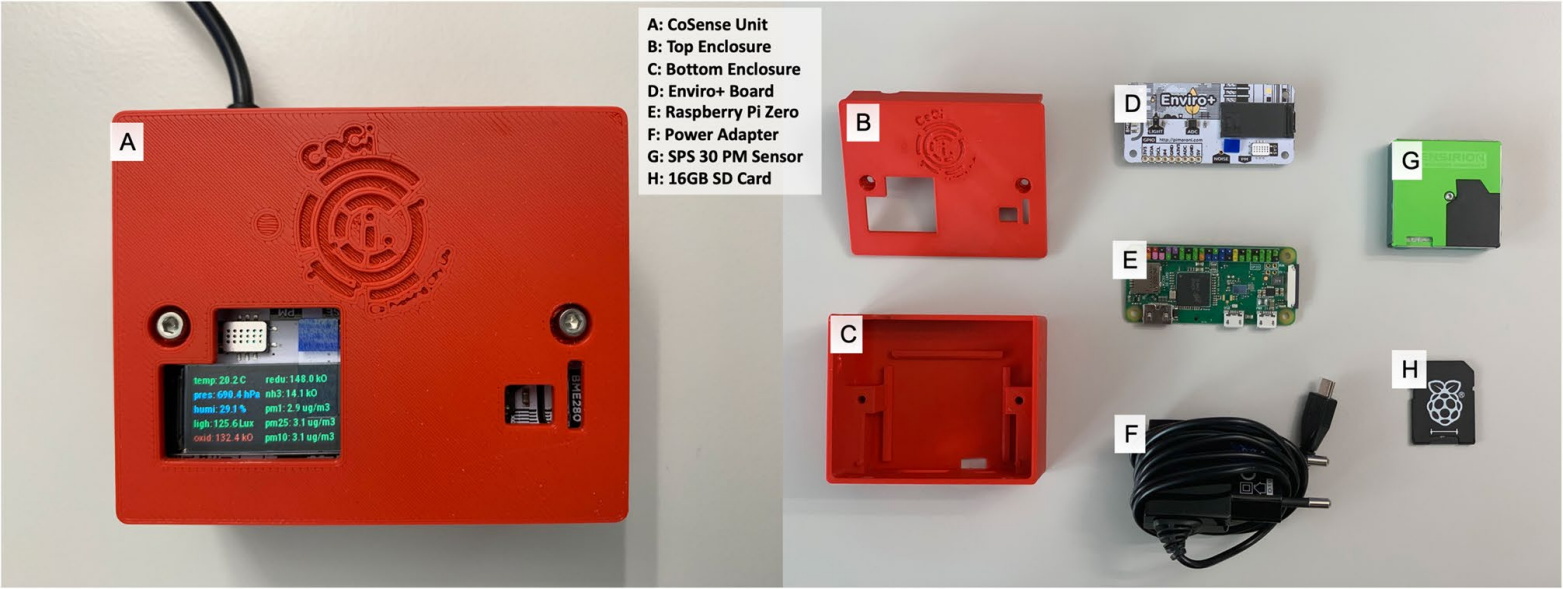
A complete and exploded view of the CoSense Unit with annotations.

Enviro Plus is particularly efficient due to its small size, seamless sensor integration, and compatibility with single board computers like Raspberry Pi. The CoSense Unit uses a Raspberry Pi Zero to communicate with the sensors using the General-Purpose Input Output (GPIO) ports. As Raspberry Pi has multiple GPIO ports, it allows flexibility to add more sensors based on the requirement of a user. Figure 4 shows the detailed view of the CoSense Unit with components and annotations. All the components are housed within a 3D-printed enclosure. The CoSense Unit is powered using a USB cable to provide a 5V supply. The users have a choice to use an adapter or a power bank for powering the Raspberry Pi. This allows the device to be used flexibly for mobile or stationary environmental monitoring.

**Figure 5:**
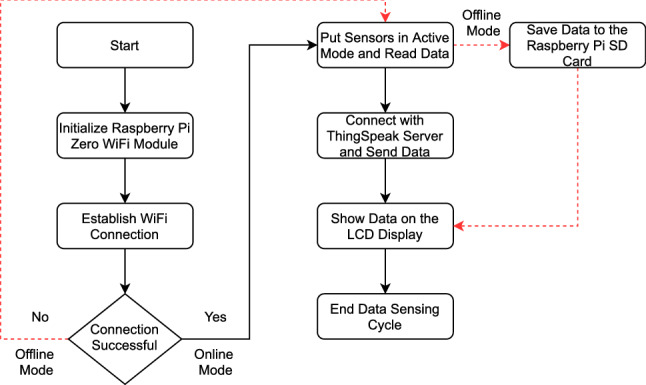
Flowchart of CoSense Unit software.

### Software implementation

The CoSense Unit uses a Raspberry Pi Zero to communicate with sensors and handles tasks related to network creation, data transmission, and storage in an SD card. Figure 5 shows the flowchart of the CoSense Unit source code. The CoSense Unit source code is written in Python programming language^53^ and uses standard sensor libraries to communicate with the sensors. As shown in Fig. 5, once the Raspberry Pi is powered on, it goes into the set-up mode. The Wi-Fi module of the Raspberry Pi goes into the Access Point (AP) mode and allows the user to connect to the device’s Wi-Fi network. Once this connection is successful, the users are redirected to a web interface that allows them to connect to a secure Wi-Fi network. The device automatically saves the Wi-Fi credentials that allow the device to connect to the saved Wi-Fi network in case of a reboot. In case no Wi-Fi network is available, the device goes into offline mode. In either case, the sensors are put in active mode following the connection test. The sensors stay awake for 30 seconds and do the measurement. The measured data is stored in the Raspberry Pi’s SD card in CSV format. When the device is in an online mode, an HTTP connection is created and the measured data is sent to the ThingSpeak server using the GET request. Once the acknowledgment is received from the server, the connection is closed. To secure the data transmission, private keys are generated by ThingSpeak before a data stream can be created. The LCD screen shows the data values from the sensors. The availability of online and offline modes allows continuous sensing of data. It is also useful in case environmental monitoring needs to be done in a remote location without internet connectivity. The current version of the prototype measures data every 5 minutes and goes to sleep mode after the measurement. The users can change the sampling frequency based on their needs.

## Results and discussion

This section describes the criterion that was used to validate and evaluate the performance of the CoSense Unit, specifically focusing on PM2.5 concentration. The results are followed by a discussion to understand how the prototype works in real-life conditions. This section also looks into the design and development of the data analysis and visualization application and how the proposed setup compares with existing environmental monitoring infrastructures.

### Sensor validation

Sensor validation is a key step in the development of environmental monitoring infrastructure. There are different ways to perform quality assurance and control of a sensing unit. This study followed a standard approach for validating the sensor by looking at the inter-sensor variability and comparing the sensor output with the official air quality monitoring station^54–56^.

**Figure 6:**
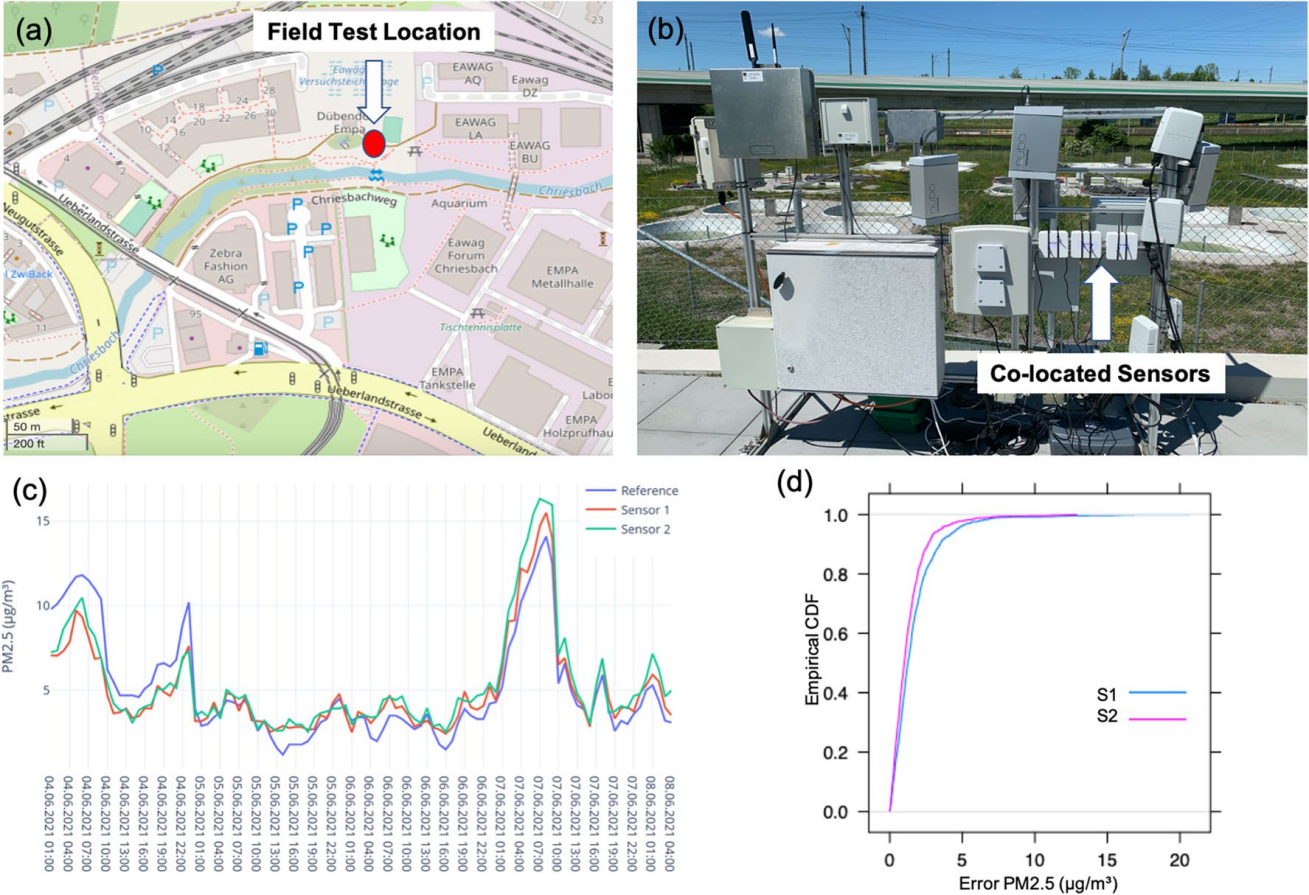
(**a**) Red dot on the map shows the field test location, (**b**) Co-location setup at NABEL monitoring station, (**c**) Line plot of PM2.5 data obtained from two CoSense Units located with the reference monitor at NABEL station, and (**d**) CDF of the difference between the PM2.5 values recorded by the reference monitor and two sensors (S1 and S2).

*Field co-location* During the summer of 2021, two CoSense Units were tested in the field in Zurich, Switzerland. To analyze the accuracy of the sensors and evaluate the performance, two units were collocated at one of the sites of the National Air Pollution Monitoring Network (NABEL). NABEL monitors air quality at 16 sites in Switzerland. For this study, the sensor units were collocated at the NABEL station in Dubendorf. Figure 6a shows the location of the test site. Figure 6b shows the actual setup of CoSense Units for colocation at the NABEL reference monitoring station. The station is located in a suburban location. The area is densely populated with a network of heavily used roads and railway lines. The field test was conducted between 4 June 2021 and 8 June 2021. The PM2.5 was sampled every five minutes and it was averaged for 1 h to maintain consistency with the PM2.5 data obtained from the reference monitor. Overall, the data was compared for 100 h. Figure 6c presents a line plot that compares the data obtained from two CoSense Units (denoted by Sensor 1 and Sensor 2) and the reference monitor. It can be observed that the CoSense Units can match the variations recorded by the reference monitor. This highlights that the CoSense Unit can successfully capture sudden variations in PM2.5 concentration in a real-world environment. The average error between the PM2.5 recorded by the reference monitor and Sensor 1 was 1 $\mu \mathrm{g}/\mathrm{m}^3$. In the case of Sensor 2, it was 1.2 $\mu \mathrm{g}/\mathrm{m}^3$.

The error value is very low and shows high accuracy and reliability of the data sensed by the CoSense Units. Figure 6d shows the empirical cumulative distribution function (CDF) to understand the PM2.5 measurement offset between the reference monitor and the two sensors. It can be observed that more than 85% of the observations have an offset below 5 $\mu \mathrm{g}/\mathrm{m}^3$. A statistical summary of the co-located data is presented in Table 1. The statistical parameters show strong similarity between the data obtained from the reference monitor and two CoSense Units.

**Table 1:** Summary statistics of PM2.5 (g/$\mathrm{m}^3$) values recorded by the reference station and two CoSense units.

	Reference PM2.5	Sensor 1 PM2.5	Sensor 2 PM2.5
Mean	5.09	5.04	5.43
Standard Error	0.31	0.27	0.31
Median	3.95	3.99	4.42
Standard Deviation	3.12	2.75	3.10
Sample Variance	9.75	7.55	9.57
Minimum	1.20	2.43	2.54
Maximum	14.1	15.5	16.32

**Figure 7:**
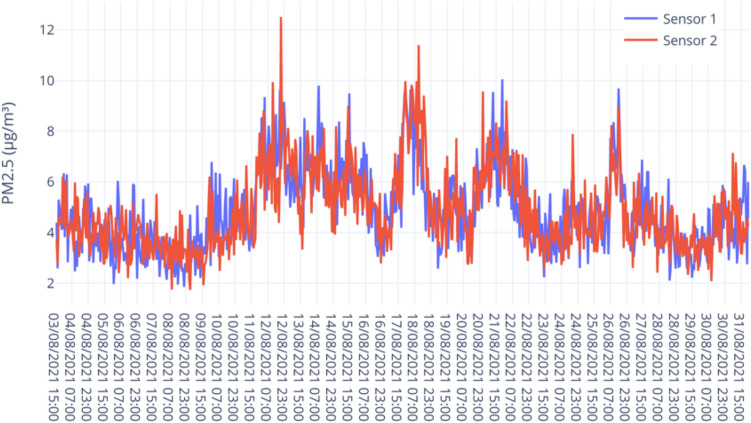
Line plot of PM2.5 data obtained from two collocated CoSense Units.

**Table 2:** Summary statistics of PM2.5 (g/$\mathrm{m}^3$) values recorded by two CoSense Units.

	Sensor1 PM2.5	Sensor2 PM2.5
Mean	4.86	4.86
Standard Error	0.06	0.06
Median	4.67	4.56
Standard Deviation	1.60	1.62
Sample Variance	2.55	2.62
Minimum	1.86	1.74
Maximum	10.05	12.51

*Inter-unit variability* Inter-unit variability is an important method to measure the similarity of data produced by the same sensor units. It is a useful metric that has been widely used to measure the data reproducibility of sensor units^40,54^. For this study, two CoSense Units were collocated and the PM2.5 data were analyzed to understand the similarity in data reported by the two units. The study was conducted between 3 August 2021 and 31 August 2021. Figure 7 shows the line plot based on the data obtained from two units. The data from both the units show a similar trend, except for some outliers. The data was sampled every 5 minutes. For analysis, the data were aggregated to hourly data. Two units were compared for a total of 681 h. As observed in Table 2, the data from the two units showed high similarity. The comparison showed similarities in the observed mean and standard error. Strong linearity was observed over the entire range of hourly averaged PM2.5 data.

*Sensor sustainability analysis* As discussed earlier in the Introduction, the environmental sustainability of IoT devices is also a critical component when discussing resource efficiency. Most sensors-related studies usually look into the power consumed by the sensors to address the environmental sustainability of low-cost sensor technology. This work looks at environmental sustainability through a different lens by examining the energy consumption of the IoT device as well as understanding the carbon footprint of the sensor code. To the best of our knowledge, there is no work within air quality monitoring literature that looks into this aspect of sensors. This can potentially help in promoting sensor code optimization as well as resource-aware IoT deployment. For this study, the focus was on two parameters: Emissions (Emissions as $\mathrm{CO}_2$-equivalents, kg of $\mathrm{CO}_2$ emitted per kilowatt-hour of electricity) and Energy Consumed (power consumed in kilowatt-hours). A CoSense Unit with a sampling frequency of 1 h would emit approximately 0.029 kg of $\mathrm{CO}_2$ for a month of regular sampling. Similarly, the energy consumption for one month’s use of the CoSense Unit would be approximately 0.072 kilowatt-hours. To put these values in context, watching Netflix for half an hour produces 0.4 kg of $\mathrm{CO}_2$^57^, and running an air purifier for 12 h would use 0.60 kilowatt-hours^58^. These values can give us an idea about how properly designed and optimized sensors can potentially be used in a sustainable way for monitoring the environment in the long run.

### Data analysis and visualization

A key part of any IoT infrastructure is an intuitive and efficient data analysis and visualization platform. IoT devices produce a massive amount of data and to make sense of such that it is important to have user-friendly platforms that can be easily used by experts as well as non-experts. Soc-IoT framework provides two options to visualize and analyze sensor data. The first option uses the in-built data analysis and visualization feature of the ThingSpeak platform. It allows the users to visualize data in real-time, create interactive graphs, set alerts, and statistically analyze the data using MATLAB functions. In addition to this, another non-sensor-specific sensor data analysis and visualization application called exploreR is proposed.

**Figure 8:**
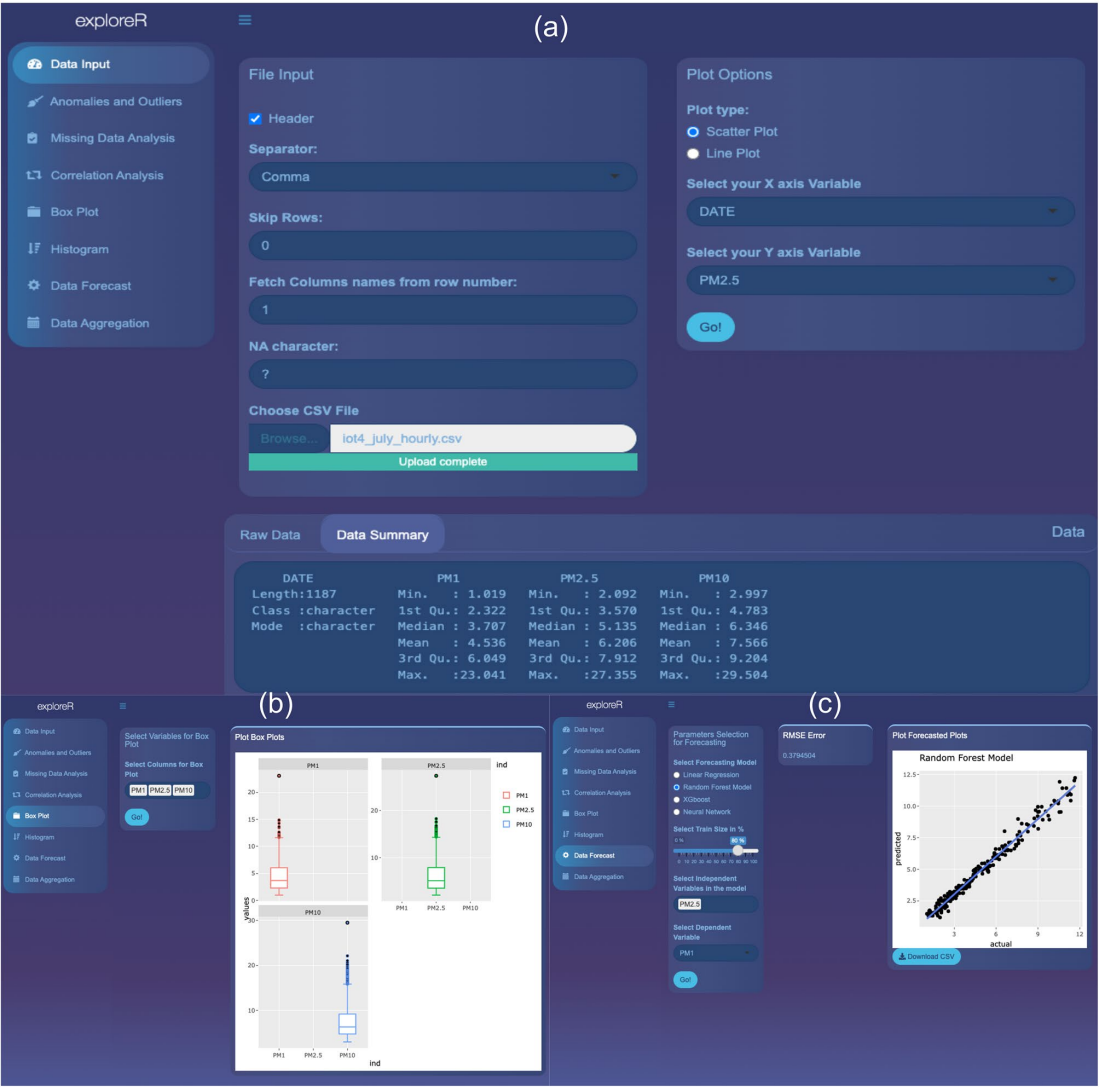
Screenshot showing some of the features of the exploreR GUI: (**a**) Landing page, (**b**) Box plot function window, and (**c**) Data forecast function window.

**Figure 9:**
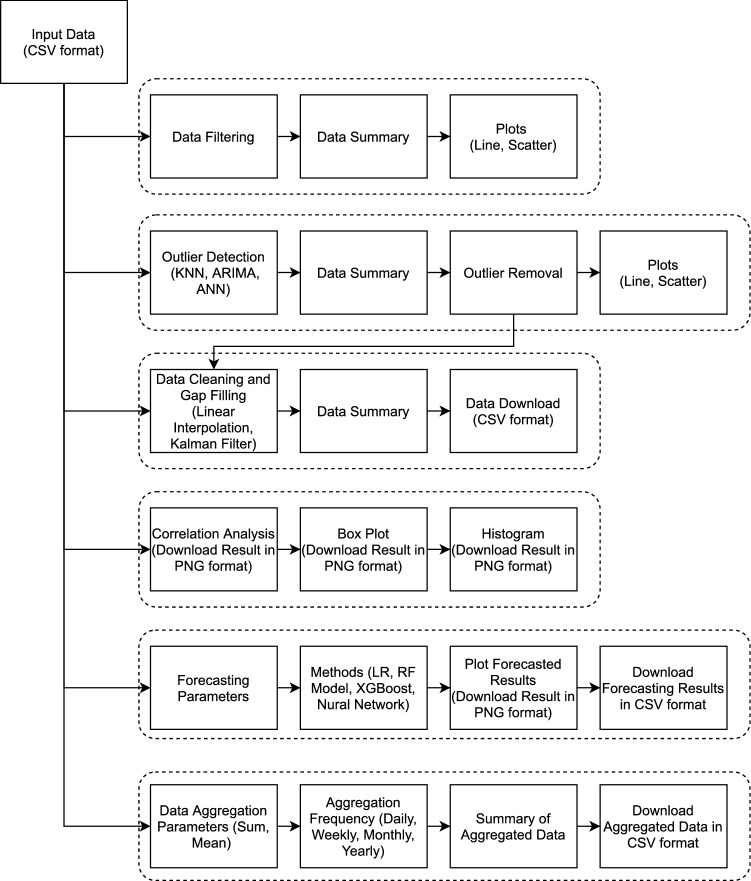
Schematic of the exploreR pipeline.

exploreR is an open-source online application that has been developed using the Shiny package in the R programming language. RShiny package has been widely used in recent years to create interactive applications for data analysis and visualizations^59–61^. Such applications have been used as a motivation to create exploreR that is designed to reduce the technical barriers especially related to coding when it comes to analyzing and visualizing citizen-generated data. The next few paragraphs explain the design and architecture of the exploreR application.

*Design and architecture* exploreR is designed as an intuitive and easy-to-use sensor data analysis and visualization. The application Graphical User Interface (GUI) is designed in a way that guides the user during the analysis process. Figure 8 shows a snapshot of the GUI of the exploreR application. The left column of the GUI (Fig. 8a) holds the main functions that expand once the user decides to use them for data analysis. Figure 8b and c shows different functions supported by the exploreR application. The application framework is designed in a way that follows a series of steps that cover the complete cycle of data input, pre-processing, visualization, and analysis. Figure 9 shows the schematic representation of the exploreR pipeline.

While designing exploreR, one of the objectives was to create an application that would facilitate usability for people from diverse backgrounds. Different integrated workflows within the application allow the user to meaningfully interpret the data without any need for coding. Here is a summary of functions supported by the current version of the application:

- Data Processing: The application accepts the data in CSV format and allows the users to filter rows/columns as well as view data summary and plot the raw data. The plots are generated using Plotly which is an interactive graphing library. The generated plots can easily be analyzed using the inbuilt functions like zoom-in/zoom-out, rescaling, among others. The users can save the generated plots in PNG format.
- Outlier Detection: The users can use sophisticated statistical and machine learning methods like k-Nearest Neighbour, ARIMA, and Artificial Neural Networks (ANN) to perform anomaly and outlier detection. Data reliability is an important topic that is widely discussed in low-cost sensor literature^55,62,63^. The outlier detection function allows the user to look for anomalies, plot them and later clean them using state-of-the-art methods.
- Gap Filling: This function allows the users to fill gaps due to missing data or gaps that are generated after removing the outliers in the previous stage. The current version of the application supports two methods: linear interpolation and Kalman filter. These methods have been used due to their widespread use in sensor literature as well as overall accuracy^64,65^.
- Exploratory Data Analysis: This feature allows the users to implement different functions on the dataset to understand the data in more detail as well observe the strengths of the relationship between different variables within the data set. The users can use the Correlation Matrix function to calculate Pearson correlation. Such information can be valuable while creating sensor calibration models^66^. The users can also create box plots and histograms to perform a visual analysis of data. The plots can be downloaded as files in PNG format.
- Data Forecasting: exploreR also has features that can be used for more advanced analysis and understanding of the air quality data. The application allows users to use advanced machine learning algorithms to perform data forecasts. PM2.5 forecast is a major challenge as has been widely studied by researchers in atmospheric science, environment monitoring, and computer science domains. The data forecast functions allow the users to use methods ranging from simple to more complex to analyze which method performs well. The current version supports methods like Linear Regression (LR), Random Forest (RF) Model, XGBoost, and ANN. The reason for selecting these models is their widespread use in time-series forecasting research^66,67^. Having multiple models allow users to compare model performance and potentially use those findings for creating real-time forecasting applications. The forecasting results can be viewed in the application as well as downloaded in CSV format.
- Data Aggregation: Different air quality sensors are programmed to record data at a different frequencies. Sometimes the data may be too granular or not granular at all. This can lead to an imbalanced time series and adversely impact the overall analysis. To address this challenge, exploreR allows the users to downsample the data to daily, weekly, monthly and yearly data. The user can either use the sum or mean to aggregate the data. The aggregated data can be downloaded in CSV format.

exploreR is a major component of the Soc-IoT framework and is aimed at the easy analysis of sensor data as well as assisting citizen scientists, policymakers, researchers from non-programming backgrounds to perform data analysis. Furthermore, exploreR facilitates the easy export of figures and files that can be used for reporting data, publications, and data dissemination.

### Comparison with existing applications

To understand how this application contributes to the field of open-source sensor data analysis, exploreR is compared with similar air quality sensor data analysis applications and softwares^61,68–71^. Different applications and softwares have been proposed over the years, with each of them having some strengths and weaknesses. Most of the applications are usually designed for the data from a specific sensor. It works well for data from particular sensors, but with data from different IoT devices, it might not work well. This is mainly due to different data formats as well as the organization of the data. Similarly, with programming-intensive tools, users who are technically experienced can easily analyze the data but it becomes difficult in case the user has no background in programming languages. Keeping these points in mind, exploreR is designed as a non-sensor-specific application that doesn’t require any prior knowledge of programming. This allows the users to analyze data from different sensors with ease and without worrying about technical complexities. At the same time, the open-source nature of the application allows the users with training in programming to improve the existing framework by using their skills to add more functions to the application.

**Table 3:** Comparison of exploreR with other air quality data analysis tools and softwares.

Name	Open Source	GUI	Sensor Specific	Programming Requirement	Data Analysis	Data Visualization	Data Forecast
OpenAir^68^	Yes	–	No	Yes	Yes	Yes	Yes
AirSensor^69^	Yes	–	Yes	Yes	Yes	Yes	No
Vayu^70^	Yes	Desktop-based	No	No	Yes	Yes	No
Data Viewer^69^	Yes	Web-based	Yes	No	Yes	Yes	No
Sense Your Data^61^	Yes	Web-based	Yes	No	Yes	Yes	No
PWFSLSmoke^71^	Yes	–	No	Yes	Yes	Yes	Yes
exploreR	Yes	Web-based	No	No	Yes	Yes	Yes

Table 3 compares exploreR with other existing open-source tools and softwares that have been widely used for analyzing air quality data obtained using low-cost sensors. Most of the existing solutions are designed keeping in mind specific sensors and user groups. The comparison highlights that exploreR successfully combines features that allow the analysis of data from different sensors without any need for programming.

### Discussion

Soc-IoT improves the accessibility to environmental data and promotes community engagement by capitalising on the recent advancements and developments of low-cost environmental monitoring sensors as well as open-source data analysis packages. It represents a novel opportunity for the citizens as well as the researchers to monitor environment using the CoSense Unit that is built using“off-the-shelf” hardware. The exploreR application on the other hand allows detailed and reproducible analysis of sensor data. Such an open-source tool can potentially bridge the gap between experts and non-experts as well as allow citizen scientists to add context while analyzing their data, which is often missing when data is evaluated by a third party. Soc-IoT has been designed as a citizen-centric platform where users can benefit from hands-on experience when it comes to using environmental monitoring sensors. The open-source nature of the framework allows for continuous development of the Soc-IoT framework while also encouraging wider community participation in environmental monitoring tasks. The methodology used for CoSense Unit validation is representative of widely used quality assurance and quality control methods for low-cost sensors. Despite the challenges of using low-cost sensors, the CoSense Unit performed well in terms of data quality when compared to data from official air quality monitoring stations. In terms of sensor sustainability, the CoSense Unit can be utilized for resource-aware IoT deployment, which not only considers the IoT device’s energy usage but also ensures that the sensor code consumes as little energy as possible. This also opens up the possibility of supplementing the official environmental monitoring system with a low-cost environmental sensing framework. As highlighted in a recent study^72^, technological complexity and limited interaction between key stakeholders are some of the key barriers to participatory citizen science. The modular and transparent nature of Soc-IoT framework allows it to be used for participatory citizen science activities that could promote citizen engagement and allow communities and decision-makers to collaborate on major environmental issues.

## Conclusion and future work

Leveraging the growth in the IoT and its interplay with sustainable practices and open-source principles, this paper proposes Soc-IoT, a proof-of-concept framework for citizen-centric environmental monitoring. The framework promotes accurate and efficient environmental monitoring by integrating open-source hardware and software. The CoSense Unit is built with readily available low-cost hardware components that can be used by researchers, citizens, and the maker community to create their own sensing devices. Because of the ease of access and low cost of these hardware components, the CoSense Unit can also be used in locations that have limited resources and budget for environmental monitoring. The performance and accuracy of the CoSense Units is extensively evaluated by co-locating them at an official air quality monitoring station equipped with reference-equivalent instrumentation in Dubendorf, Switzerland. Additionally, quality assurance was performed by studying the inter-unit variability. With a modular design, easy assembly, and intuitive data analysis interface, the Soc-IoT framework can assist in air pollution exposure assessment as well as comprehensive analysis of air quality data. The exploreR application is designed to reduce technical barriers, particularly those related to programming. It offers both experts and non-experts a wide range of data analysis and visualization functionalities that support visual inspection of data, data cleaning, and detailed data analysis.

The core part of the framework focuses on enhancing embedded spatial intelligence where citizen empowerment meets smart environments and sustainable design.The proposed framework has the potential to foster collaboration among a wide range of stakeholders, including scientists, policymakers, and citizens and maker community. The CoSense Unit’s reliability and accuracy enable it to potentially complement official environmental monitoring networks. The extensible and open-source nature of Soc-IoT framework would encourage others to use it as a development platform rather than reinventing everything from scratch. To strengthen the science-policy-society interface, the Soc-IoT framework can also be used to facilitate co-creation and Citizen Science activities. Besides supporting data democratization, it can be used to create an environment where citizens’ opinions, observations, and expertise are valued and used to facilitate a dialogue with decision-makers.

The framework presented in this paper demonstrates the feasibility of using open-source low-cost IoT technology in environmental monitoring applications. Therefore, the work presented here can be used for future research. Although the environmental sustainability of IoT devices has been considered as part of this work, there are other aspects that could not be considered due to time constraints, such as the social and economic sustainability of IoT. Future work will look into the social and economic viability of the technological solutions discussed in this paper. Another future research direction would be to investigate data-related issues such as user data security and privacy, as well as to evaluate various privacy preservation techniques to protect user data. In order to improve the system’s scalability, future research will also look into dynamic calibration and edge analytics. Additional enhancements will be made to the data analysis tool, including improvement in the user interface and the addition of more functionalities. A key part of the future works would include conducting field experiments in collaboration with the research as well as the citizen science community to analyze the usability of the device for promoting environmental awareness.

## Data Availability

The sensor code, STL files for 3D printing as well as the code for explorR application are available freely at Github https://github.com/sachit27/Soc-IoT. The exploreR application can be accessed using this link https://sachitmahajan.shinyapps.io/exploreR/.
